# Study on plastic flow of conditioned soil within pressure chamber of deeply buried EPB shields tunneling through sandy stratum

**DOI:** 10.1038/s41598-026-43016-7

**Published:** 2026-03-10

**Authors:** Xiaochun Zhong, Siyuan Huang, Haijun Wang, Qiang Yun

**Affiliations:** 1 College of Applied Engineering, Gandong University, Fuzhou, 344000 Jiangxi China; 2 College of Civil and Transportation Engineering, Hohai University, Nanjing, 210098 Jiangsu China; 3 China Railway Construction South China Construction Co., Ltd, Guangzhou, 511458 Guangdong China

**Keywords:** EPB shield tunneling, Soil conditioning, Soil discharge, Bingham fluid, Slump test, Engineering, Environmental sciences, Solid Earth sciences

## Abstract

For earth pressure balance (EPB) shield tunneling, the stability of tunnel face is controlled by the excavation and discharge rates of soil within tunnel pressure chamber. To ensure continuous discharge of soil from pressure chamber, the soil is required to have favorable plastic flow. However, the plastic flow of conditioned soil primarily relies on experience that lacks relevant theoretical guidance. Additionally, when EPB shields are used for tunneling in deeply buried sandy strata, common soil conditioners may struggle to make the conditioned soil have ideal plastic flow. By assuming the conditioned soil as an ideal Bingham fluid, a simplified soil slump model and a calculated model for passive soil discharge are developed in this study to assess the plastic flow of conditioned soil using yield stress and dynamic viscosity coefficient. By incorporating macromolecular polyacrylamide (PAM) along with fine particles, the conditioned soil could have favorable plastic flow at any slump. Based on the measured soil discharge rate from Hengli - Panyu Square Station of Guangzhou Metro Line 18, the ideal plastic flow and slump of conditioned soil under various tunnel burial depths are derived. The results indicate that as the tunnel burial depth increases, the yield stress of the conditioned soil must be systematically increased, and consequently, the slump must be reduced to ensure the integrity of the earth plug and anti-spewing safety. This study provides a systematic inversion methodology for determining optimal soil conditioning parameters based on burial depth, offering a theoretical framework and safety thresholds for the adaptive management of soil conditioning in deep-buried EPB shield tunneling.

## Introduction

Earth pressure balance (EPB) shield tunneling is widely employed in urban subway construction. In recent years, the overall construction trend of EPB shield tunneling has developed towards complex strata such as large burial depth and high-water pressure^1–9^. By adjusting the speed of the screw conveyor (i.e., soil discharge rate), the soil pressure in pressure chamber can be controlled to ensure the stability of tunnel face and thus to minimize disturbance to surrounding environment^10^. If tunnels are constructed in cohesionless strata (i.e., sand or sand-pebble ground) with a large burial depth, it is challenging to control the soil discharge rate based on existing experience. This challenge may lead to large fluctuations of supporting pressures and increase the risk of collapse at the excavation surface. Therefore, to accurately control tunnel face supporting pressure and gain a reasonable soil discharge rate, it is necessary to explore the plastic flow of conditioned soil within pressure chamber of shield tunnel under various burial depths^11–13^.

**Fig. 1:**
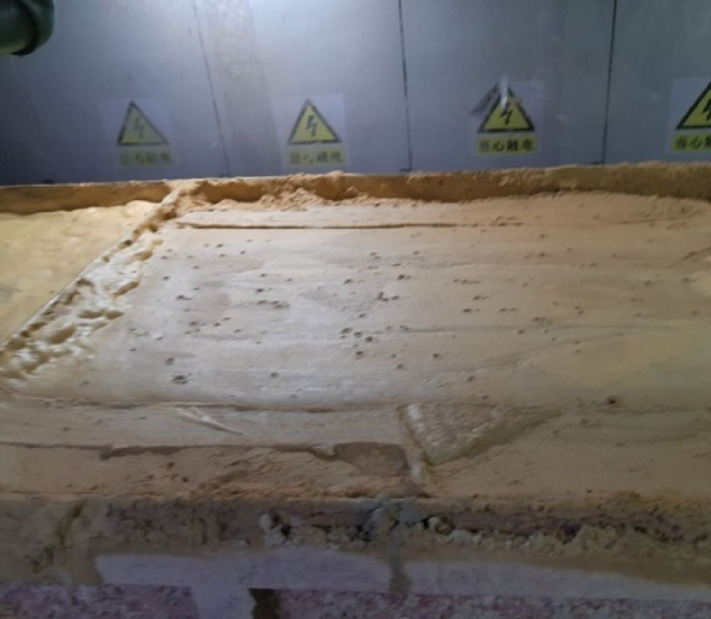
Conditioned soil in toothpaste-like state.

One of the key technologies in EPB shield tunneling is soil conditioning. In practice, common soil conditioners such as foam, bentonite slurry, dispersant and flocculant were used to change soil properties within pressure chamber^14^. For tunnels constructed in complex geological ground conditions, the conditioned soil within pressure chamber should have appropriate plastic flow to ensure a reasonable soil discharge rate. In practice, slump test is commonly used as an evaluation index for plastic flow of conditioned soil^14^. The ideal state of the conditioned soil should be similar to toothpaste^14,15^, as shown in Fig. 1. Currently, a series of soil conditioning methods has been developed to facilitate the construction of EPB shield tunneling. By adding foam into pelitic siltstone within pressure chamber, Ye et al.^16^ found that soil discharge efficiency during tunnel construction was greatly improved and thus the thrust force-torque of the shield tunnel was significantly reduced. Based on the field measurement, the ideal slump of conditioned soil was suggested in a range of 170–200 mm. For a tunnel constructed in Shenzhen gravelly sand stratum with a burial depth ranging from 14 to 16m, Qiu et al.^17^ found that the usage of 8–10% bentonite slurry (i.e., a bentonite to water ratio is 1:7) can give an ideal soil slump ranging from 195 to 210 mm. Tao et al.^18^ proposed a new foam-soil for Guangzhou Metro Line 21 constructed in completely decomposed granite with a burial depth ranging from 12 m to 22 m. For this ground condition and tunnel burial depth, the suggested ideal soil slump was in the range of 100–150 mm. Moreover, the ideal slump obtained from Quebau et al.^19^, Budach and Thewes^20^, Zhao et al.^21^ and Martinelli and Peil^22^ were 120 mm, 100–200 mm, 205–210 mm, and 150–200 mm, respectively. Obviously, the ideal slump is greatly affected by soil stratum and tunnel burial depth. This is because the plastic flow of conditioned soil is influenced by several factors, such as soil properties, soil conditioner types, and excavation parameters.

In summary, the existing ideal slump range for conditioned soil is primarily determined through subjective experience. It is generally required that the slump of the conditioned soil remains within a range of 100–200 mm; however, this range is too broad. As the burial depth of shield tunneling increases, the experience-based slump may become less applicable. The ideal slump of conditioned soil may further decrease with increasing burial depth of the tunnel, potentially dropping below 100 mm ^23^. At this stage, improving the soil to achieve an ideal plastic flow using only soil conditioners such as foam, water, and bentonite slurry becomes increasingly difficult. Therefore, in order to ensure that the slump of the conditioned soil is suitable for the burial depth of the tunnel and that the soil has favorable plastic flow, it is essential to study the correlation between plastic flow of conditioned soil and burial depth to guide on-site soil conditioning.

Therefore, this paper addresses the aforementioned issues through the following works: (1) The conditioned soil is assumed to behave as an ideal Bingham fluid, and its plastic flow is quantified based on rheological parameters; (2) The incorporation of macromolecular polyacrylamide (PAM) combined with fine particles as the soil conditioner ensures that the conditioned soil retains favorable plastic flow even at lower slump values.; (3) Based on the monitoring data of soil discharge from Guangzhou Metro Line 18 and utilizing the theoretical formula for soil discharge rate, we inverted the on-site soil plastic flow. This approach can be extended to other burial depth conditions to derive reasonable soil plastic flow.

## Study on the plastic flow of conditioned soil

### Relationship between slump and rheology of conditioned soil

**Fig. 2:**
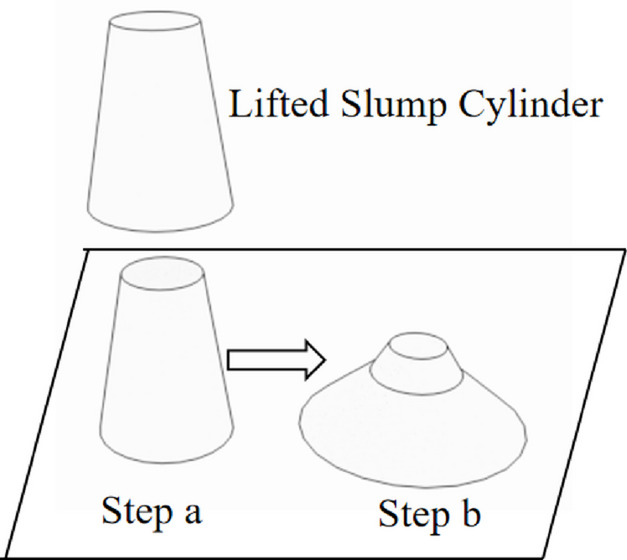
Slump test.

**Fig. 3:**
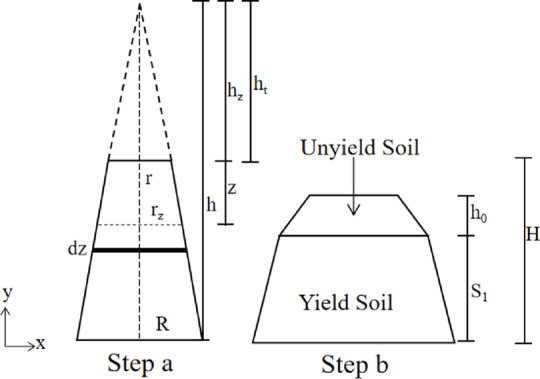
Mechanical model of soil slump test.

By using a cone mechanical model, Murate^24^ investigated the relationship between yield stress and slump of filling slurry. By modifying Murate’s model, Christensen^25^ proposed a dimensionless research approach to estimate soil slump. In this section, a simplified slump mechanical model using current research theory is proposed. The assumptions are listed as follows : (1) Conditioned soil is considered as an ideal Bingham fluid; (2) The soil within slump cylinder is incompressible. The slump test comprises two main processes: (a) the slump cone is lifted completely away from the soil, at which point the soil has not collapsed; (b) the soil collapses entirely. As shown in Fig. 2, the collapse of the soil during the slump test occurs from the bottom to the top. This is because the pressure at the bottom is greater, causing soil yielding at these regions. Conversely, the unyielded part must exist in the upper half of the soil. To further simplify the model, the following soil slump mechanical model is proposed, as depicted in Fig. 3. The model essentially calculates the variation in height caused by vertical gravitational stress exceeding yield stress, neglecting the complex lateral spreading friction to obtain a simplified analytical solution.

The conditioned soil often exhibits non-linear rheological behavior such as shear-thinning, which is more accurately described by the Herschel-Bulkley (H-B) model. However, in this study, the conditioned soil is considered as an ideal Bingham fluid for several critical reasons:

Dominance of Yield Stress: For the sandy stratum conditioned to a “toothpaste-like” state, the stability of the tunnel face and the formation of the earth plug are primarily governed by the yield stress. In the low-to-medium shear rate ranges typical of EPB operations, the linear approximation of the Bingham model effectively captures this threshold behavior.

Mathematical Tractability: The objective of this study is to establish a direct, closed-form analytical relationship between the slump (S) and rheological parameters. The Bingham model allows for an explicit derivation that site engineers can easily apply, whereas the H-B model introduces non-linear exponents that significantly complicate the inverse analysis without providing a proportional increase in practical accuracy for site-level conditioning.

Engineering Conservatism: At higher shear rates within the screw conveyor, the Bingham model tends to overestimate the shear stress compared to a shear-thinning model. For “Anti-Spewing” safety assessments, this provides a conservative margin, ensuring that the predicted flow resistance is not underestimated.

Validity Range: The Bingham assumption is considered valid for the conditioned sandy muck within the shear rate range experienced during standard screw conveyor rotations and static slump tests. Previous studies on conditioned sand^22^ have successfully utilized Bingham parameters to characterize EPB muck workability, supporting the reliability of this simplification for engineering practice.

As shown in Fig. 3, the z-th layer (dz) of soil is subjected to static pressure from the soil above the z-th layer (dz), as shown in Eq. (1):

1 $${P_{\mathrm{z}}}=\frac{{\rho {\mathrm{g}}{V_{\mathrm{z}}}}}{{\pi {\mathrm{r}}_{{\mathrm{z}}}^{2}}}=\frac{{\rho {\mathrm{g}}}}{3}({{\mathrm{h}}_{\mathrm{z}}}{\mathrm{-}}\frac{{{{\mathrm{r}}^2}}}{{{\mathrm{r}}_{{\mathrm{z}}}^{2}}}{{\mathrm{h}}_{\mathrm{t}}})$$

Where *P*_*z*_ is the static pressure exerted on the soil in layer z, *ρ* is the soil density, *g* is the acceleration of gravity, *V*_z_ is the volume of the soil above layer z.

Based on the geometric relationship described by Eq. (2), Eq. (1) can be transformed into Eq. (3):

2 $$\frac{{{{\mathrm{h}}_{\mathrm{t}}}}}{{\mathrm{r}}}=\frac{{H+{{\mathrm{h}}_{\mathrm{t}}}}}{R}{\text{ }}\frac{{{{\mathrm{h}}_{\mathrm{z}}}}}{{{{\mathrm{r}}_{\mathrm{z}}}}}=\frac{{H+{{\mathrm{h}}_{\mathrm{t}}}}}{R}{\text{ }}\frac{{{{\mathrm{h}}_{\mathrm{t}}}}}{{\mathrm{r}}}=\frac{{{{\mathrm{h}}_{\mathrm{z}}}}}{{{{\mathrm{r}}_{\mathrm{z}}}}}{\text{ }}$$

3 $${P_{\mathrm{z}}}=\frac{{\rho {\mathrm{g}}}}{3} \cdot \frac{{\mathrm{r}}}{{R{\mathrm{-r}}}}\left[ {{\mathrm{1}}+\frac{{\mathrm{z}}}{{\mathrm{H}}} \cdot \frac{{R{\mathrm{-r}}}}{{{r}}}{\mathrm{-}}\frac{1}{{(1+\frac{{\mathrm{z}}}{H} \cdot \frac{{R{\mathrm{-r}}}}{{\mathrm{r}}}){ ^2}}}} \right]$$

Based on the Tresca yield criterion, the shear stress (*τ*_*z*_) of the z-layer soil can be obtained as shown in Eq. (4):

4 $${\tau _{\mathrm{z}}}=\frac{1}{2}{P_{\mathrm{z}}}=\frac{{\rho {\mathrm{g}}H}}{6}\left[ {1+\frac{{\mathrm{z}}}{H}{\mathrm{-}}\frac{1}{{{{(1+\frac{{\mathrm{z}}}{H})}^2}}}} \right]$$

**Fig. 4:**
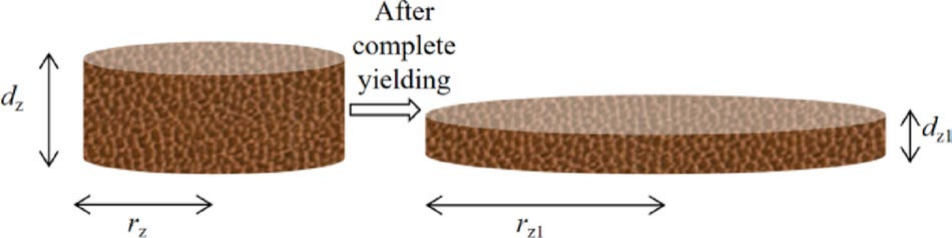
microsegment of soil *d*_z._

As shown in Fig. 3, the soil layer undergoes yielding deformation when the shear stress exceeds the yield stress of the soil. The flow stops once the shear stress equals the yield stress, at which point the height of the slump is defined as the ‘slump’. Based on the previously mentioned assumption of the incompressibility of the soil, the volume of the yielding soil micro-segment remains constant. Consequently, the micro-segment of soil ( *d*_z_ ) at stage a and the micro-segment ( *d*_z1_ ) at stage b satisfy the Eq. (5), as illustrated in Fig. 4.

5 $${{\mathrm{d}}_{{\mathrm{z}}1}}=\frac{{{\mathrm{r}}_{{\mathrm{z}}}^{2}}}{{{\mathrm{r}}_{{{\mathrm{z1}}}}^{2}}}{{\mathrm{d}}_{\mathrm{z}}}{\text{ }}{\tau _{\mathrm{z}}}=\frac{{M{\mathrm{g}}}}{{2\pi {\mathrm{r}}_{{\mathrm{z}}}^{{\mathrm{2}}}}}{\text{ }}{\tau _{{\mathrm{z1}}}}={\tau _{\mathrm{0}}}=\frac{{M{\mathrm{g}}}}{{2\pi {\mathrm{r}}_{{\mathrm{z}}}^{{\mathrm{2}}}}}{\text{ }}$$

Where *M* is the mass of the soil in the *d*_z_ layer.

By integrating the micro-segments of the yielding soil, the height of the yielding soil can be calculated, referred to as the ‘deformation part’ in this study. The height of the ‘deformed part’ is presented in Eq. (6).

6 $${S_1}=\int_{{{{\mathrm{h}}_0}}}^{H} {{\text{ d}}{{\mathrm{z}}_{\mathrm{1}}}} \int_{{{{\mathrm{h}}_0}}}^{H} {{\text{ }}\frac{{{\tau _{\mathrm{0}}}}}{{{\tau _{\mathrm{z}}}}}{\text{ dz}}=} \int_{{{{\mathrm{h}}_0}}}^{H} {{\text{ }}{\tau _{\mathrm{0}}}\frac{{\mathrm{6}}}{{\rho {\mathrm{gH}}}}\frac{{\mathrm{1}}}{{(1+\frac{{\mathrm{z}}}{H}) \cdot \frac{1}{{{{(1+\frac{{\mathrm{z}}}{H})}^2}}}}}{\text{ dz}}} =\frac{{2{\tau _0}}}{{\rho {\mathrm{g}}}}{\mathrm{ln}}\left[ {\frac{7}{{{{(1+\frac{{{{\mathrm{h}}_0}}}{H})}^3}{\mathrm{-}}1}}} \right]$$

Where *S*_1_ is the height of ‘deformed part’.

For the undeformed segment, the yield stress of the soil is equal to the shear stress. Therefore, the height of the undeformed segment is shown in Eq. (7):

7 $${\tau _0}=\frac{{\rho g{\mathrm{H}}}}{6}\left[ {1+\frac{{{h_0}}}{H} - \frac{1}{{{{(1+\frac{{{h_0}}}{H})}^2}}}} \right]$$

Where $h_{0}$ is the height of ‘undeformed part’.

Therefore, the soil slump is defined as follows:

8 $$S=H-S_{1}-{h_{0}}$$

Where *S* is the soil slump. Since the conditioned soil is assumed to be behave as an ideal Bingham fluid, its plastic flow can be characterized by rheological parameters, as shown in Eq. (9):

9 $$\tau ={\tau _0}+\mu \frac{{{\mathrm{du}}}}{{{\mathrm{dy}}}}$$

Where *τ* is the shear stress of the soil, *τ*_0_ is the yield stress of the soil, *µ* is the dynamic viscosity coefficient, $\frac{{{\mathrm{du}}}}{{{\mathrm{dy}}}}$ is the shear rate. From Eq. (6) to (8), it can be seen that the slump of soil is only related to its own yield stress.

### Verification test

**Fig. 5:**
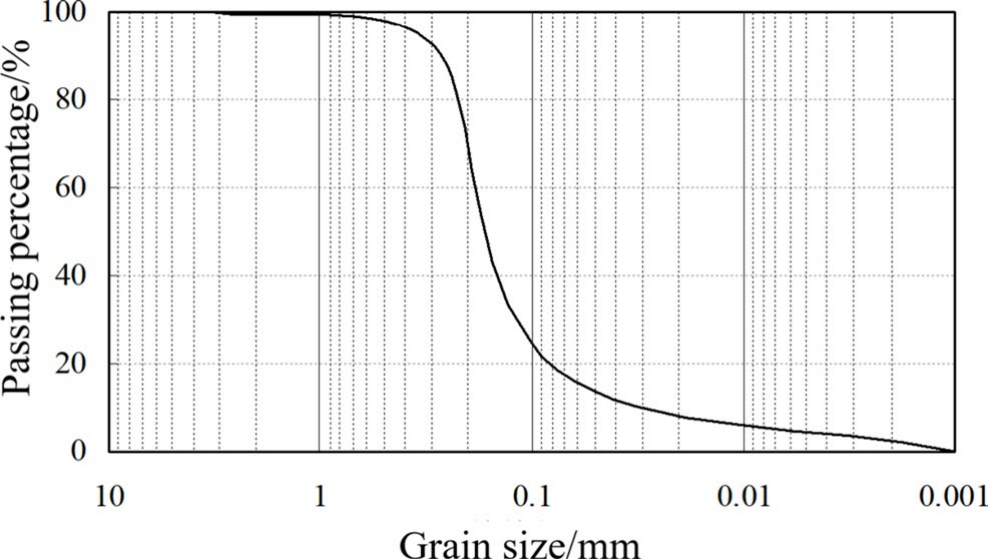
Particle size distribution of the soil in the test.

To verify the proposed slump mechanical model, the rheological parameters of the soil were tested by using the NXS-11B rotary viscometer. Theoretical slump was compared with the experimental slump to validate the mechanical model. The primary soil conditioners used in the test were foam and bentonite slurry. The bentonite slurry was prepared with a ratio of 1:4 (bentonite to water) and fermented for 8 h after thorough mixing. The concentration of the foaming agent mixture was 3%, with a foaming rate of 20 times. The soil used in the tests primarily consists of fine sand, with a density of 1.585 g/cm³. The particle size distribution is illustrated in Fig. 5, and the soil conditioning plan is presented in Table 1. The ‘content’ in the Table 1 is the quality ratio of soil conditioner to soil. FIR in the Table 1 represents the injection ratio of foam, and is the ratio of the volume of foam mixed to the volume of the soil.

**Table 1:** Plans of soil conditioning.

ID	Water content(%)	Bentonite slurry content(%)	FIR(%)
S1	10	17	50
S2	10	17	45
S3	10	17	35
S4	10	17	25
S5	10	17	15

**Fig. 6:**
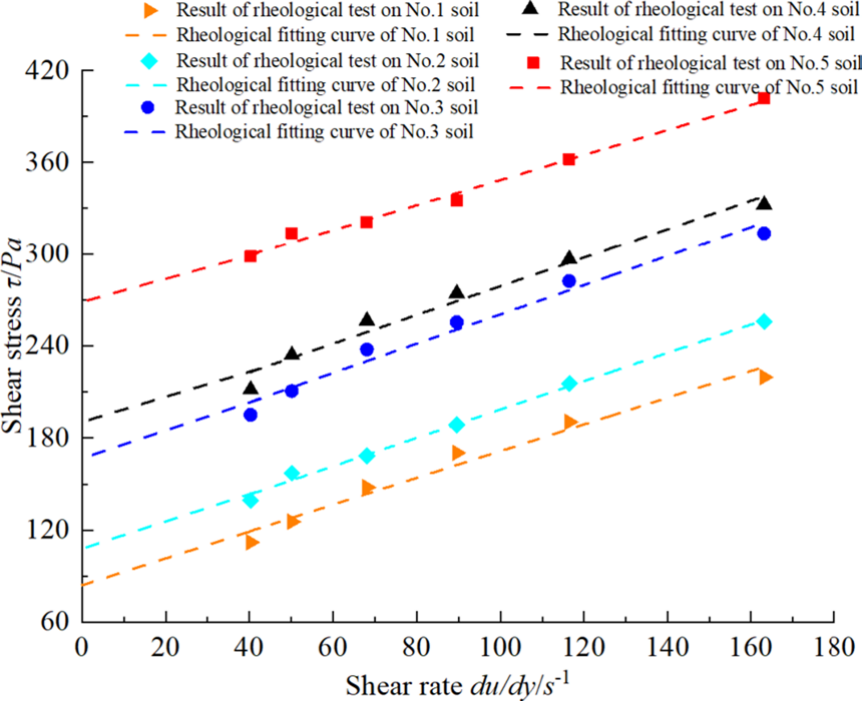
Rheological parameters of the conditioned soil in test.

All conditioned soils were tested for density and rheological parameters to calculate the theoretical slump. Assuming that the conditioned soil behaves as an ideal Bingham fluid, the rheological curve should satisfy Eq. (9). Consequently, finally the rheological parameters of the soil can be derived, as shown in Fig. 6. The soil densities of all groups are 1.526 g/cm^3^, 1.589 g/cm^3^, 1.597 g/cm^3^, 1.601 g/cm^3^and1.603 g/cm^3^.

**Fig. 7:**
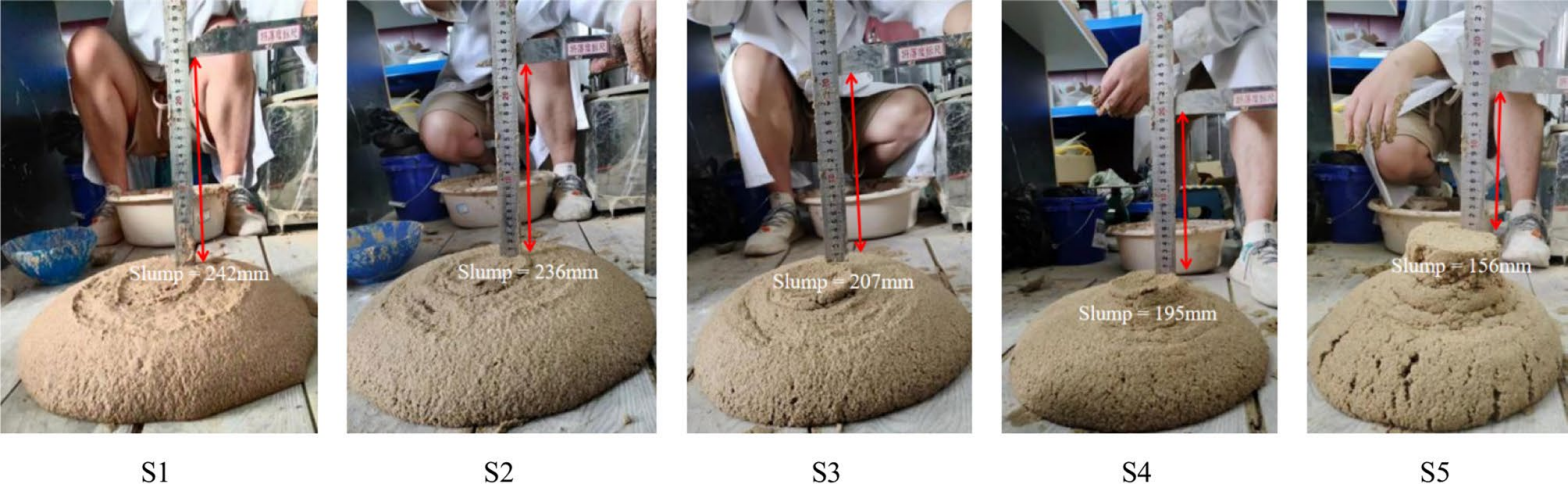
Slump tests.

From Fig. 6, the yield stress and the dynamic viscosity coefficients of conditioned soils can be obtained. 1 # soil: *τ*_0_ = 84.311 Pa, *µ* = 0.871 Pa·s, 2 # soil: *τ*_0_ = 106.121 Pa, *µ* = 0.924 Pa·s, 3 # soil: *τ*_0_ = 165.082 Pa, *µ* = 0.954 Pa·s, 4 # soil: *τ*_0_ = 185.2541 Pa, *µ* = 0.937 Pa·s, 5 # soil: *τ*_0_ = 266.121 Pa, *µ* = 0.817 Pa·s. It can be observed from Fig. 6 that as the FIR increases, the yield stress of the conditioned soil significantly decreases. This reduction occurs because foam acts as a ‘lubricant’ in the soil conditioning due to its ball effect. As the FIR increases, the plastic flow of the soil also improves, leading to an increase in the slump. The yield stress obtained from the tests were substituted into Eqs. (6), (7), and (8) to calculate the theoretical slump for each soil group, while the experimental slump were measured, as presented in Table 2; Fig. 7.

**Table 2:** Theoretical slump and experimental slump.

FIR(%)	Theoretical slump value/mm	Experimental slump value/mm	Errors/%
50	243.525	242	0.63
45	234.396	236	0.67
35	207.981	207	0.47
25	199.802	195	2.46
15	169.364	156	8.57

Table 2 indicates that when the FIR exceeds 45%, the soil has a strong plastic flow and a large slump, and the theoretical slump is basically consistent with the experimental slump. However, as the FIR decreases, the errors between the theoretical slump and experimental slump increases. When the FIR is 15% the measured and calculated slumps is differed by 8.57%. As the slump decreases, the conditioned soil may no longer behave as a Bingham fluid. Figure 7 illustrates that the surface of Soil 5 (FIR = 15%) exhibits “dry cracking”, indicating it has poor plastic flow. Consequently, it is challenging to make conditioned soil remain favorable plastic flow under low slump state using solely foam and bentonite slurry as soil conditioner.

### PAM blended fine particles for soil conditioning

**Fig. 8:**
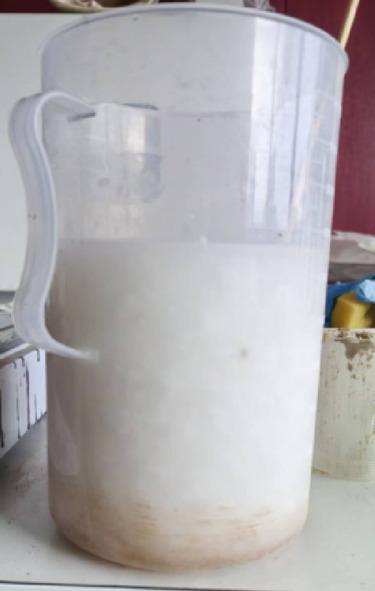
PAM solution.

**Fig. 9:**
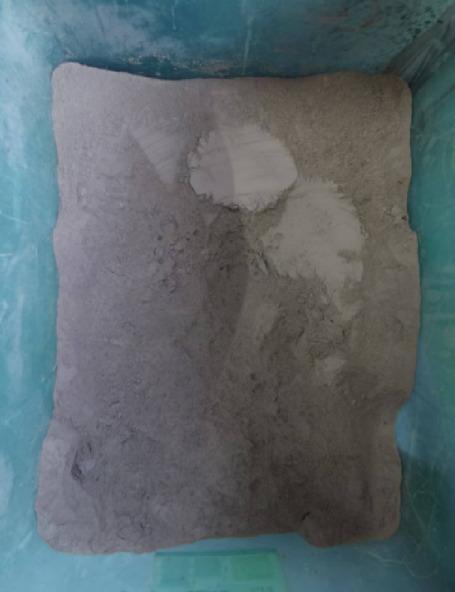
Fine particles.

Polymers are a class of macromolecules formed by the covalent bonding of one or more molecules or molecular clusters (structural units or monomers) containing multiple repeating monomer units^26,27^. When polymers are added to the soil, they bond with the surfaces of the soil particles through physical adsorption or chemical bonding, resulting in a stable composite material that forms a three-dimensional network structure. This structure facilitates stress dispersion and enhances the workability of the soil^28–31^. We utilize a macromolecular polyacrylamide (PAM) as the soil conditioner, supplemented by fine particles. The PAM solution was prepared in a mass ratio of 1:14, combining the original PAM solution with 500 mL of 50℃ distilled water. The PAM solution is illustrated in Fig. 8, the fine particles are primarily composed of powder particles, which are depicted in Fig. 9, with a particle size range of 0.01 to 0.07 mm. These fine particles are added to the original sand sample in a mass ratio of 1:14. The bentonite slurry was prepared with a ratio of 1:4 (bentonite to water) and fermented for 8 h after thorough mixing. The concentration of the foaming agent mixture was 3%, with a foaming rate of 20 times. The PAM solution and sand samples were mixed as three groups of conditioned soil, labeled a, b, and c, at mass ratios of 1:10, 1:12, and 1:15.

**Fig. 10:**
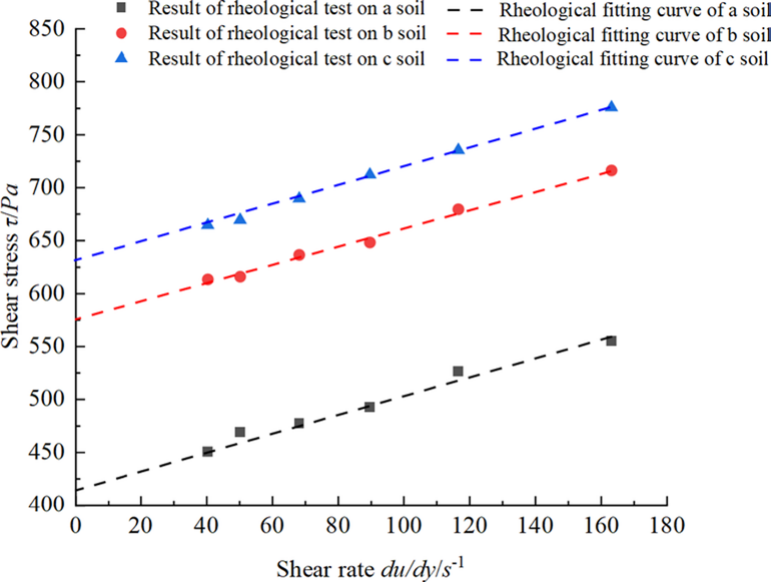
Rheological results of PAM conditioned soil.

**Fig. 11:**
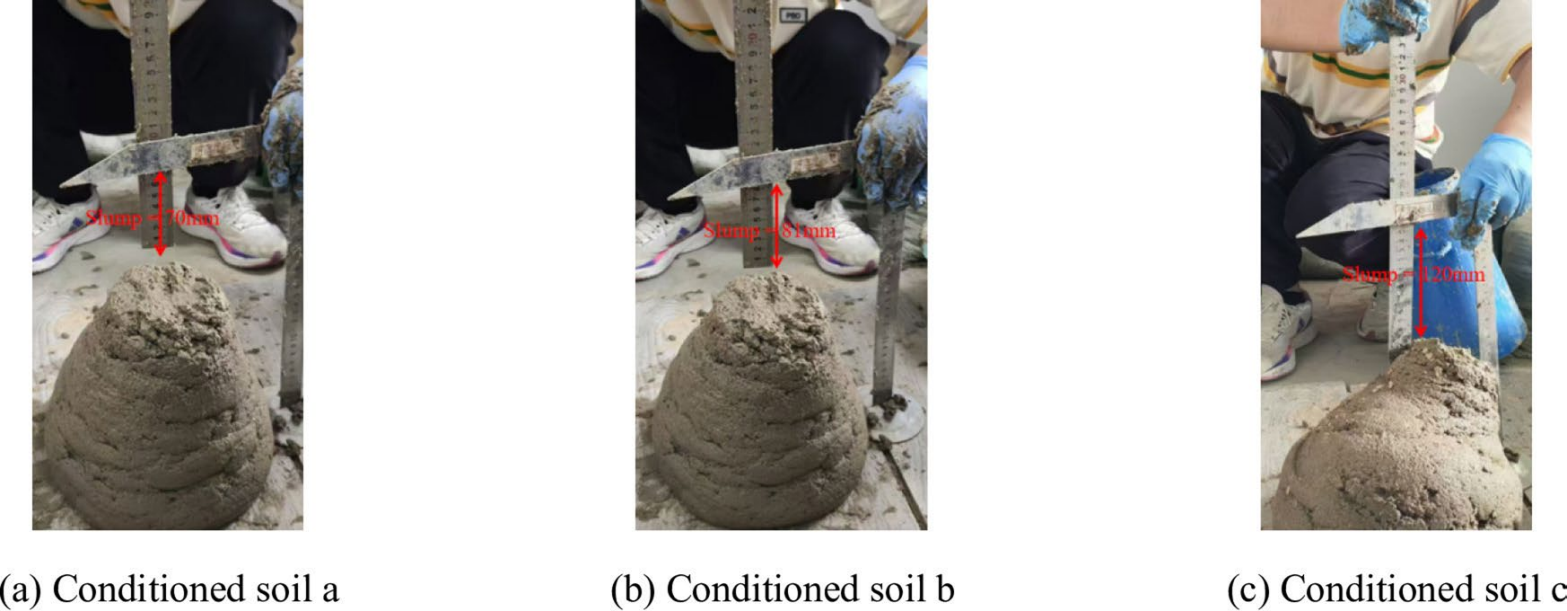
Slump test of PAM conditioned soil.

The conditioned soils a, b, and c exhibit favorable plastic flow. From Fig. 10, it is evident that the conditioned soil treated by the incorporation of PAM and fine particles meets the rheological characteristics of Bingham fluids. The test results indicate that the density of conditioned soil a is 1.651 g / cm^3^, with rheological parameters of *τ*_0_ = 421.2 Pa and *µ* = 0.852 Pa·s; the density of conditioned soil b is 1.691 g / cm^3^, with rheological parameters of *τ*_0_ = 575.2 Pa and *µ* = 0.871 Pa·s; and the density of conditioned soil c is 1.713 g / cm^3^, with rheological parameters of *τ*_0_ = 626.1 Pa and *µ* = 0.904 Pa·s. Substituting these values into Eqs. (6), (7), and (8) yields slump values of 121.08 mm, 83.23 mm, and 72.58 mm, respectively. The actual slump values obtained from slump tests are 120 mm, 81 mm, and 70 mm, as shown in Fig. 11. The errors for the slump are relatively small, at 0.9%, 2.7%, and 3.7%, respectively. This indicates that utilizing the PAM and fine particle as soil conditioners ensures that the conditioned soil have favorable plastic flow even under low slump conditions, and its properties effectively meet the characteristics of Bingham fluids.

**Fig. 12:**
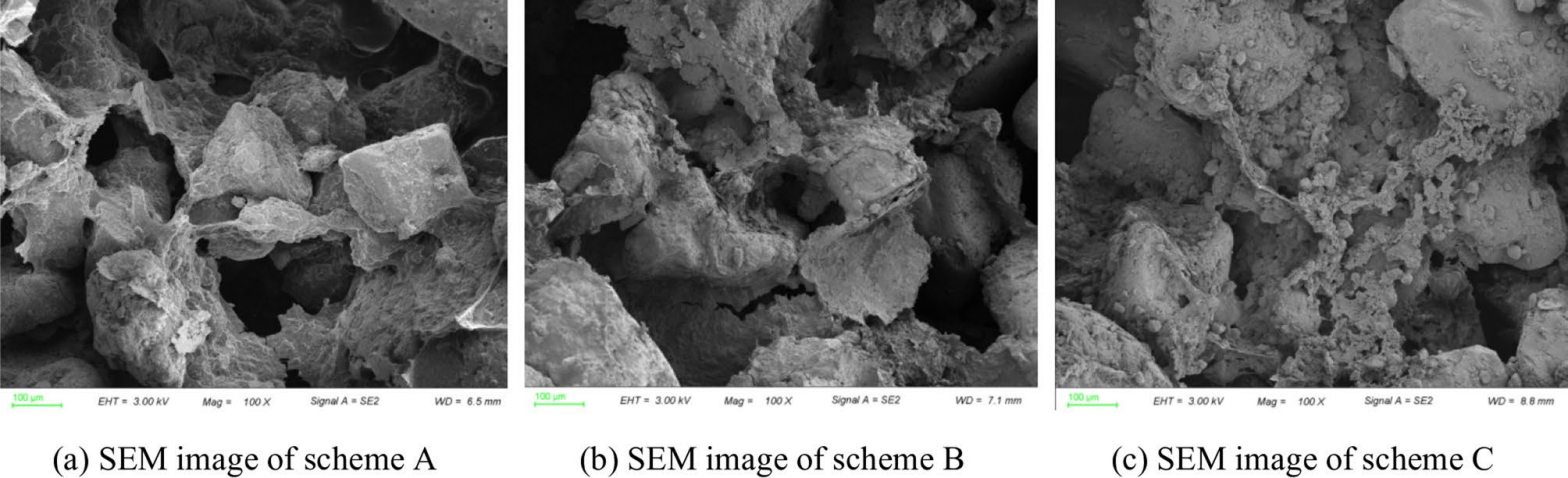
SEM images.

Scanning Electron Microscopy (SEM) was employed to observe the microscopic characteristics of the conditioned soil with PAM blended fine particles as soil conditioners and to further analyze its conditioned mechanism. To study the joint conditioned mechanism of PAM and fine particles on soil, two new conditioned schemes were introduced as control groups: Scheme A (conditioner: water + bentonite slurry + PAM + foam), Scheme B (conditioner: water + bentonite slurry + foam), and Scheme C (modifier: water + bentonite slurry + PAM + foam + fine particles). The properties of these conditioners are consistent with those described in the previous section. The SEM images of the conditioned soil are presented in Fig. 12.

Figure 12(a) illustrates that PAM forms a clear three-dimensional network. The polymer chains adsorb onto particle surfaces, creating inter-particle “bridges” that bond fine and coarse grains together. This flocculated structure increases the energy threshold required for the soil to initiate flow, which macroscopically manifests as a significant increase in yield stress. In contrast, Scheme B (Fig. 12b) shows a loose particle distribution; without polymer bridging, the bentonite slurry alone cannot provide sufficient cohesion to prevent structural collapse under high pressure. As depicted in Fig. 12(c), the introduction of fine particles ensures a more compact matrix by filling the macroscopic voids between coarse sand grains. These fine particles, coated with PAM flocs, act as “lubricating bearings”. Once the yield stress is exceeded, these polymer-encapsulated fine flocs facilitate grain-to-grain sliding, reducing internal friction between large particles. This mechanism allows the soil to maintain a relatively stable dynamic viscosity despite the increase in *τ*_0_.

Consequently, the synergy of fine-particle filling and PAM bridging enables the muck to maintain superior stability (high *τ*_0_) and manageable pumpability (stable *µ*), forming an ideal earth plug that is critical for deep-buried EPB shield tunneling under high hydrostatic pressure.

## Study on passive discharge model under pressure constraint

This paper considers the discharge of conditioned soil with ideal plastic flow, modeled as a Bingham fluid. In practice, the screw conveyor regulates soil discharge through its rotational speed to balance soil inflow and outflow, thereby stabilizing the face support pressure. However, under high face pressure, the soil may flow through the conveyor driven solely by the pressure gradient, even when the screw is not rotating. This model characterizes the hydraulic conductivity of the screw conveyor under pressure gradients, representing the critical state for preventing spewing when the screw is stationary or assisting the pressure balance, rather than the active mechanical transport.

Consequently, this paper analyzes the soil discharge mechanism under this critical state by omitting the influence of rotational speed. This approach allows for the determination of the rheological thresholds required to maintain a stable “earth plug.” To establish the mathematical model, the following assumptions are made: (1) The rotational speed of the screw conveyor is not considered to focus on the pressure-driven limit; (2) The ideal conditioned soil is an incompressible and isotropic Bingham fluid; (3) The flow state of conditioned soil in the screw conveyor is assumed to be laminar flow; (4) The conditioned soil is assumed to maintain constant parallel straight flow in the screw conveyor.

### Mechanical model of soil discharge

**Fig. 13:**
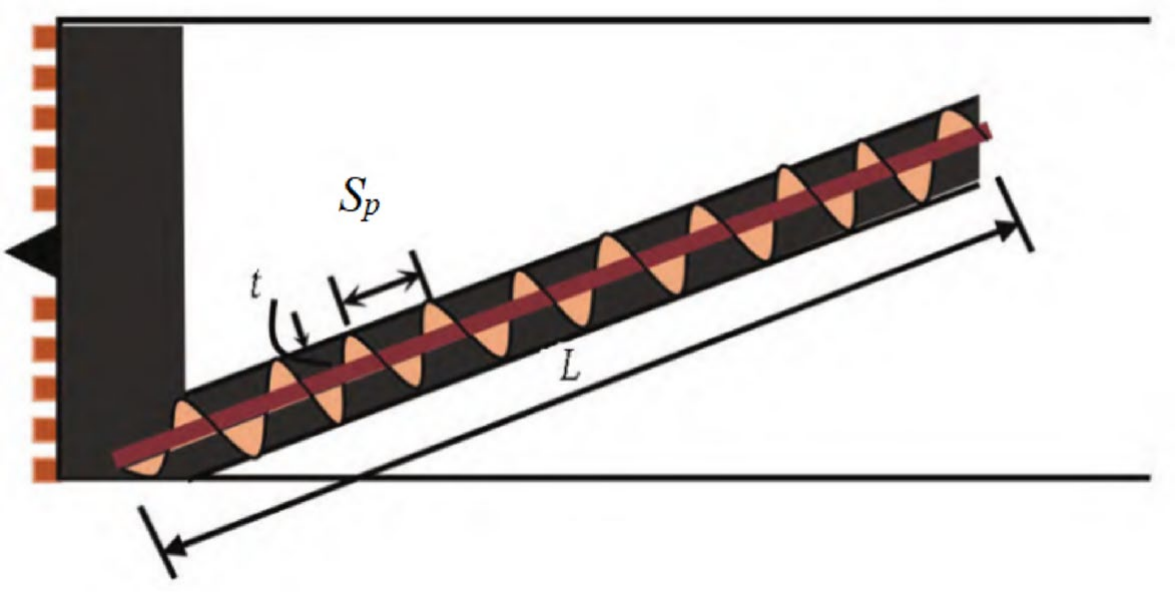
Schematic diagram of the structure of screw conveyor.

**Fig. 14:**
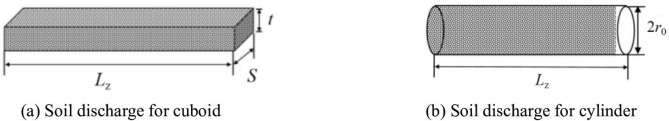
Unfolded bodies of screw conveyor. (**b**) Soil discharge for cylinder.

The structure of the screw conveyor is illustrated in Fig. 13. The cross-section of the screw conveyor in the discharge direction is rectangular. Since the speed of the screw conveyor is not taken into account, the screw conveyor can be unfolded along the discharge direction to obtain a cuboid discharge path. The unfolded diagram of the screw conveyor is presented in Fig. 14. The length of the unfolded discharge path is calculated using Eq. (10):

10 $${L_Z}=\left( {L/{S_p}} \right) \cdot \sqrt {{{(\pi D)}^2}+{S_p}^{2}}$$

Where *L* is the length of the screw conveyor, *S*_*p*_ is the length of the screw pitch, and *D* is the inner diameter of the screw conveyor. In Fig. 13, t is the distance from the inner edge of the screw conveyor to the outer edge of the central shaft.

To simplify the calculation of soil discharge, the unfolded cuboid illustrated in Fig. 14 is treated as a cylinder. This approach ensures that the cross-sectional areas of the two unfolded bodies remain unchanged, thereby maintaining a constant soil discharge. Consequently, the cross-sectional radius of the cylinder satisfies the following equation:

11 $${{\mathrm{r}}_{\mathrm{0}}}=\sqrt {\frac{{{S_p}{\mathrm{t}}}}{\pi }}$$

Where *r*_0_ is the radius of the cylindrical unfolded body.

**Fig. 15:**
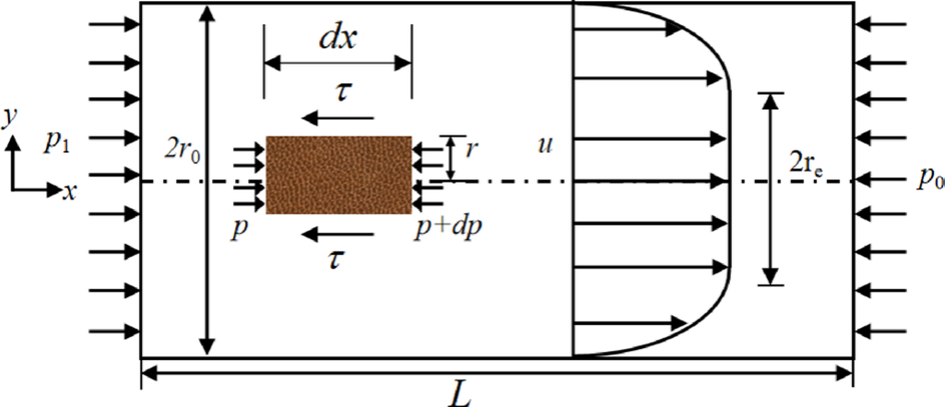
Schematic diagram of soil flow in the screw conveyor.

The motion of Bingham fluid in a circular tube are illustrated in Fig. 15. The conditioned soil flows under the pressure applied at the inlet of the tube. A unit body *dx* is considered along the length in the x-direction of the circular tube, where the decrease in soil pressure (dp) in the x-direction is counteracted by the shear resistance generated on the cylindrical surface with a radius (r). Consequently, the stress balance relationship for the unit can be expressed as shown in Eq. (12):

12 $$(\pi {{\mathrm{r}}^2}){\mathrm{dp}}=2\pi rdx\tau \Leftrightarrow \tau =\frac{r}{2}\frac{{dp}}{{dx}}$$

In Eq. (12) and Fig. 15, *P*_0_ is the end pressure, *P*_1_ is the starting edge pressure, *r* is the radius of the unit body, *r*_e_ is the radius of flow core, *r*_0_ is the radius of the cylindrical unfolded body, *τ* is the shear stress, and *u* is the flow velocity of the conditioned soil.

Bingham fluid has a flow core during its flow process, where the *du/dy* = 0 within the flow core. Consequently, the flow velocity of the conditioned soil within the flow core is uniform and represents the maximum flow velocity. The flow velocity gradually decreases across the cross section from the edge of the flow core to the edge of the circular tube. According to the no-slip boundary condition, when the radius of the flow core *r*_e_ is equal to the radius of the cylinder *r*_0_, the flow of the conditioned soil will stop. At this point, the shear stress generated by the pressure cannot overcome the yield stress of the soil, resulting in the inability of the soil to flow. Therefore, the yield stress and flow core radius of the conditioned soil must satisfy the following equation:

13 $${\tau _0}=\frac{{{r_{\mathrm{e}}}}}{2}\frac{{dp}}{{dx}} \Leftrightarrow {r_{\mathrm{e}}}=2{\tau _0}\frac{{dx}}{{dp}}({r_{\mathrm{e}}}<{r_{\mathrm{0}}})$$

Substituting Eqs. (12) and (13) into Eq. (9), the velocity distribution of conditioned soil in the *y -* direction can be obtained, as shown in Eq. (14):

14 $$\frac{{d{\mathrm{u}}}}{{dr}}=\frac{1}{{2\mu }}\frac{{dp}}{{dx}}(r - {r_e})$$

From Eq. (14), the flow velocity of the conditioned soil at any point outside the flow core can be obtained. Subsequently, Eq. (14) is integrated along the *y-*direction from *r*_e_ to *r.* By applying the boundary condition *r* = *r*_0_, *u* = 0, the flow velocity of the conditioned soil outside the flow core [*r*_e_, *r*_0_] can be derived:

15 $${{\mathrm{u}}_0}=\frac{1}{{4\mu }}\frac{{dp}}{{dx}}(r_{0}^{2}+2{r_e}r - 2{r_e}{r_0} - {r^2})$$

In the flow core [0, *r*_e_], the flow velocity of conditioned soil can be obtained by substituting *r* = *r*_e_ into Eq. (15):

16 $${{\mathrm{u}}_{\mathrm{e}}}=\frac{1}{{4\mu }}\frac{{dp}}{{dx}}{({r_0} - {r_e})^2}$$

Based on the flow characteristics of Bingham fluid, the total flow rate of the conditioned soil in the circular tube comprises two components: the flow rate *Q*_0_ within the flow core zone and the flow rate *Q*_1_ in the velocity gradient zone. Therefore, the total flow rate *Q* can be expressed as *Q* = *Q*_0_+*Q*_1_, and their calculation formulas are as follows:

17 $${Q_0}=\pi {\mathrm{r}}_{{\mathrm{e}}}^{2}{{\mathrm{u}}_{\mathrm{e}}}=\frac{\pi }{{4\mu }}\frac{{{\mathrm{dp}}}}{{{\mathrm{dx}}}}{\mathrm{(r}}_{{\mathrm{0}}}^{{\mathrm{2}}}{\mathrm{r}}_{{\mathrm{e}}}^{2}{\mathrm{-}}2{{\mathrm{r}}_0}{\mathrm{r}}_{{\mathrm{e}}}^{3}+{\mathrm{r}}_{{\mathrm{e}}}^{4})$$

18 $$\begin{gathered} {Q_1}=\int_{{{{\mathrm{r}}_{\mathrm{e}}}}}^{{{{\mathrm{r}}_0}}} {{{\mathrm{u}}_{\mathrm{0}}}} \cdot 2\pi {\mathrm{rdr}} \hfill \\ =\frac{\pi }{{4\mu }}\frac{{{\mathrm{dp}}}}{{{\mathrm{dx}}}}( \frac{{{\mathrm{r}}_{0}^{4}}}{2}{\mathrm{-}}\frac{{2{\mathrm{r}}_{0}^{3}{{\mathrm{r}}_{\mathrm{e}}}}}{3}{\mathrm{-r}}_{0}^{2}{\mathrm{r}}_{{\mathrm{e}}}^{2}+2{{\mathrm{r}}_0}{\mathrm{r}}_{{\mathrm{e}}}^{3}{\mathrm{-}}\frac{5}{6}{\mathrm{r}}_{{\mathrm{e}}}^{4}) \hfill \\ \end{gathered}$$

Finally, by adding and simplifying Eqs. (17) and (18), the flow rate of the conditioned soil can be obtained:

19 $$Q=\frac{{\pi {\mathrm{r}}_{0}^{4}}}{{8\mu }}\frac{{{\mathrm{dp}}}}{{{\mathrm{dx}}}}(1{\mathrm{-}}\frac{{\mathrm{4}}}{{\mathrm{3}}}\frac{{{{\mathrm{r}}_{\mathrm{e}}}}}{{{r_0}}}+\frac{1}{3}\frac{{r_{e}^{4}}}{{r_{0}^{4}}})$$

When the pressure is high and *r*_e_ < < *r*_0_, the higher-order term of Eq. (19) can be neglected to obtain the simplified flow rate Q^*^ of the conditioned soil:

20 $${Q^ * }=\frac{{\pi {\mathrm{r}}_{0}^{4}}}{{8\mu }}\frac{{{\mathrm{dp}}}}{{{\mathrm{dx}}}}(1{\mathrm{-}}\frac{{\mathrm{4}}}{{\mathrm{3}}}\frac{{{{\mathrm{r}}_{\mathrm{e}}}}}{{{r_0}}})$$

### Verification of the theoretical model of soil discharge

It is important to clarify that the theoretical discharge model established in Eq. (19) characterizes the passive flow rate under the pressure gradient of the excavation chamber. In engineering practice, the screw conveyor is the primary mechanism for regulating face pressure; however, if the conditioned soil flows too rapidly under pressure alone (even when the screw is stationary), the operator cannot effectively control the discharge rate, leading to the risk of “spewing.” Therefore, this model essentially defines the “Anti-Spewing Safety Limit” or the “Minimum Rheological Threshold.” It determines the necessary yield stress and viscosity to ensure that the soil plug can effectively seal the chamber pressure.

Equation (19) is the formula for the discharge flow rate of conditioned soil. To verify the reliability of the above theoretical solution, a corresponding numerical model is established using Fluent for verification.

**Fig. 16:**
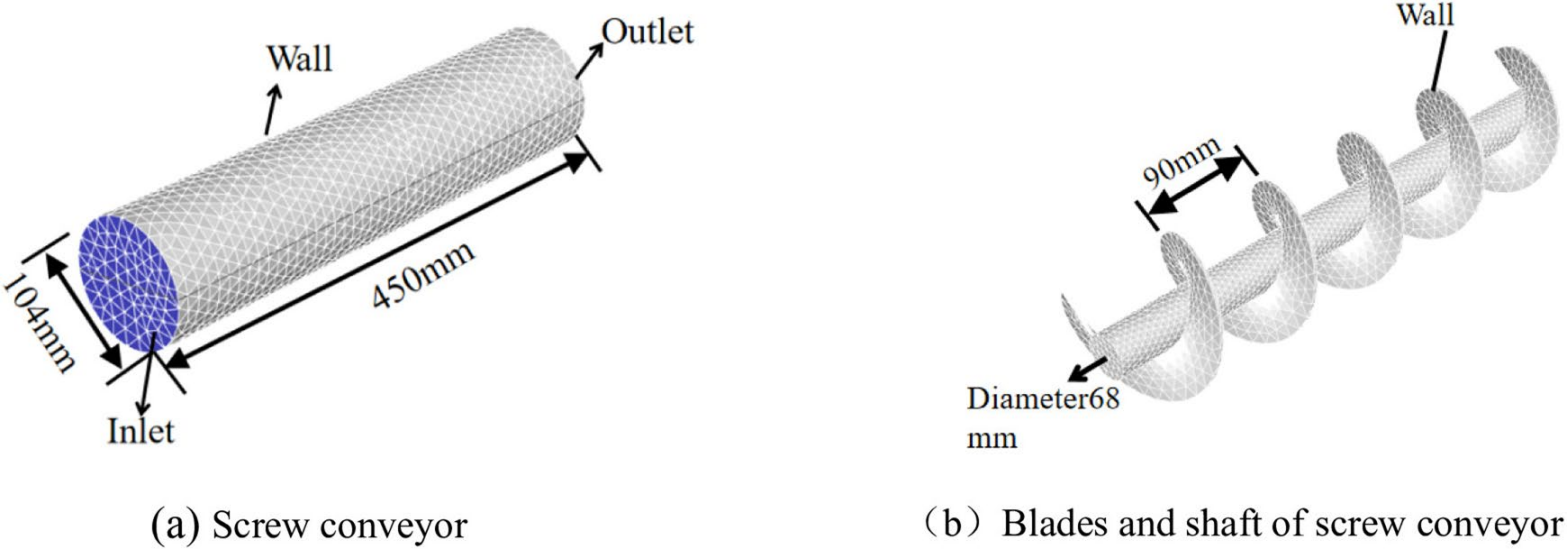
Numerical model of screw conveyor. (**b**)Blades and shaft of screw conveyor.

1) Numerical model: The established screw conveyor model is illustrated in Fig. 16. The length *L* of the screw conveyor is 450 mm, the inner diameter *D* of the screw conveyor is 104 mm, the diameter of the central shaft is 68 mm, and the screw pitch *S*_*p*_ is 90 mm. The conditioned soil in screw conveyor is divided into 68768 grids using unstructured grids. The inlet and outlet boundary conditions are set as constant pressure boundary conditions. Other components, such as the sleeves, blades, and shaft of the screw conveyor, are designated as non-inflow and no-slip wall boundaries.

2) Model parameter: The fluid constitutive model used is Herschel Bulkley rheological model (Vajravelu et al., 2005). This constitutive model is utilized in Fluent for simulating Bingham fluid, with the rheological parameters summarized in Table 2. The entire calculation is configured for steady-state analysis. The inlet pressure is set to 100 kPa and 200 kPa respectively, and the outlet pressure is set to 0 kPa.

**Fig. 17:**
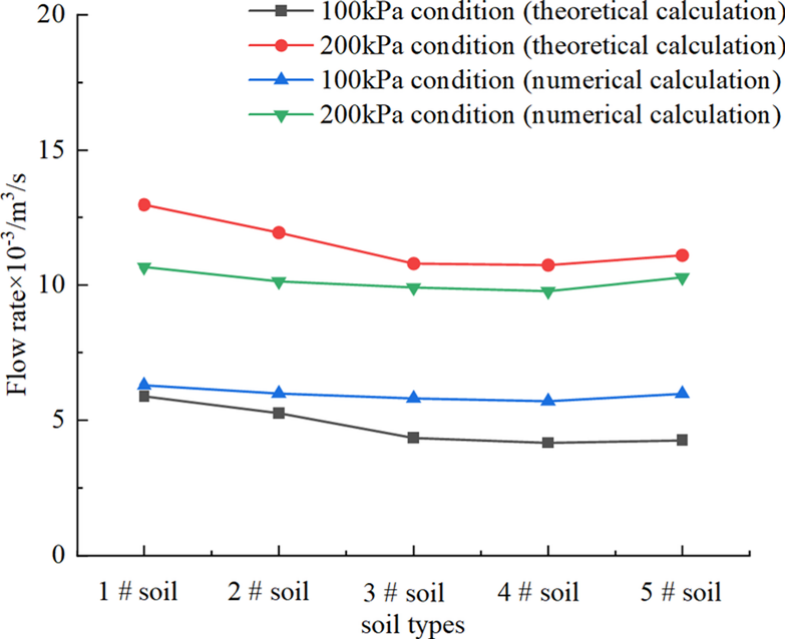
Comparison of theoretical and numerical calculations.

By comparing the theoretical and numerical calculations presented in Fig. 17, it is found that the variation trends of both calculations are generally consistent. As the inlet boundary pressure increases, the flow rate of conditioned soil increases; conversely, as the yield stress increases, the flow rate decreases. Although a maximum error of 28.76% exists, the results are largely consistent. This discrepancy primarily stems from the 1D pipe-flow simplification, which neglects the 3D internal geometry (shaft and flights) and the leakage flow through the casing clearance. Nonetheless, the established model is reliable for estimating soil discharge trends and safety thresholds in engineering practice.

## Analysis of soil discharge

**Fig. 18:**
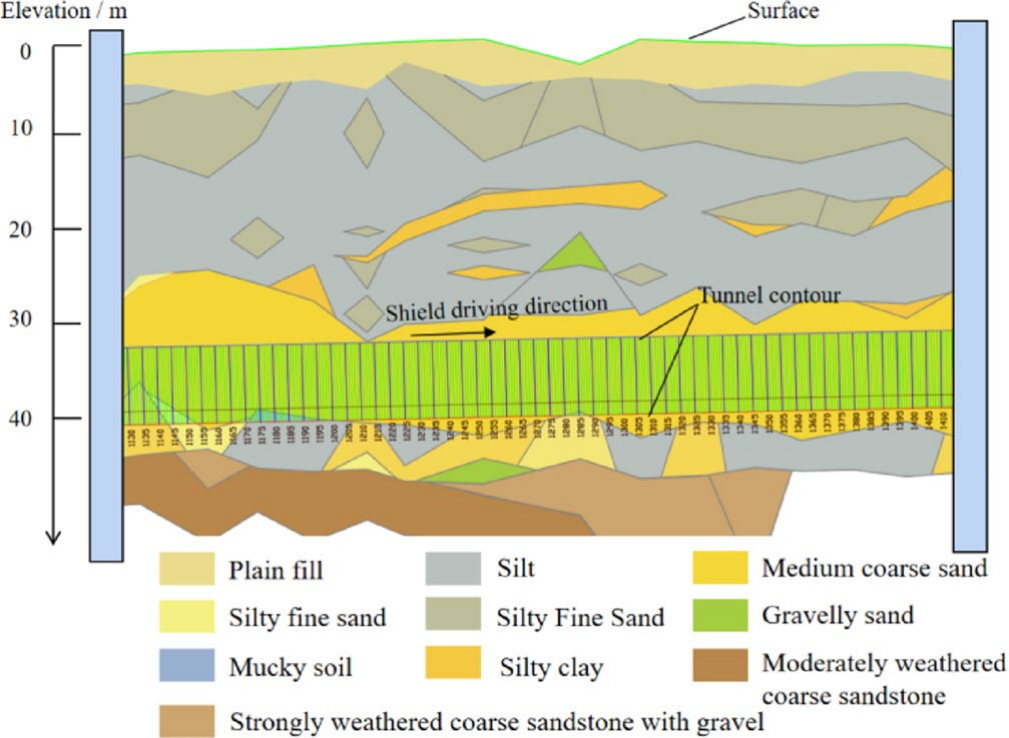
Geological section of shield tunnel crossing.

To analyze the influence of factors such as pressure and soil plastic flow on the soil discharge. We use the shield tunneling construction of the section between Hengli Station and Panyu Square Station on Guangzhou Line 18 as the engineering background. The main strata crossed by the right line include medium coarse sand, muddy soil, fine sand, moderately weathered conglomerate, slightly weathered conglomerate, and strongly weathered granite. Geological longitudinal sections are shown in Fig. 18. The burial depth of the tunnel vault is approximately 33.2 m, and the monitoring pressure in the pressure chamber is about 300 kPa. The engineering utilized EPB shield machine with a diameter of 8.8 m for excavation, which maintained a fast excavation speed, typically ranging from 20 to 30 min per ring (corresponding to an advance speed of approximately 53–80 mm/min). The width of the segment is 1.6 m. Considering the coefficient of looseness, the theoretical soil discharge for excavating one ring is about 100 m³, which is equivalent to a flow rate of 0.0556 to 0.0833 m³/s. This indicates that the engineering requires that the soil discharge for excavation of one ring should be controlled within the range of 0.0556 ~ 0.0833m^3^/s. The inner diameter of the screw conveyor is 1.02 m, the length of screw conveyor is 13.321 m, and the diameter of screw shaft is 0.285 m.

**Fig. 19:**
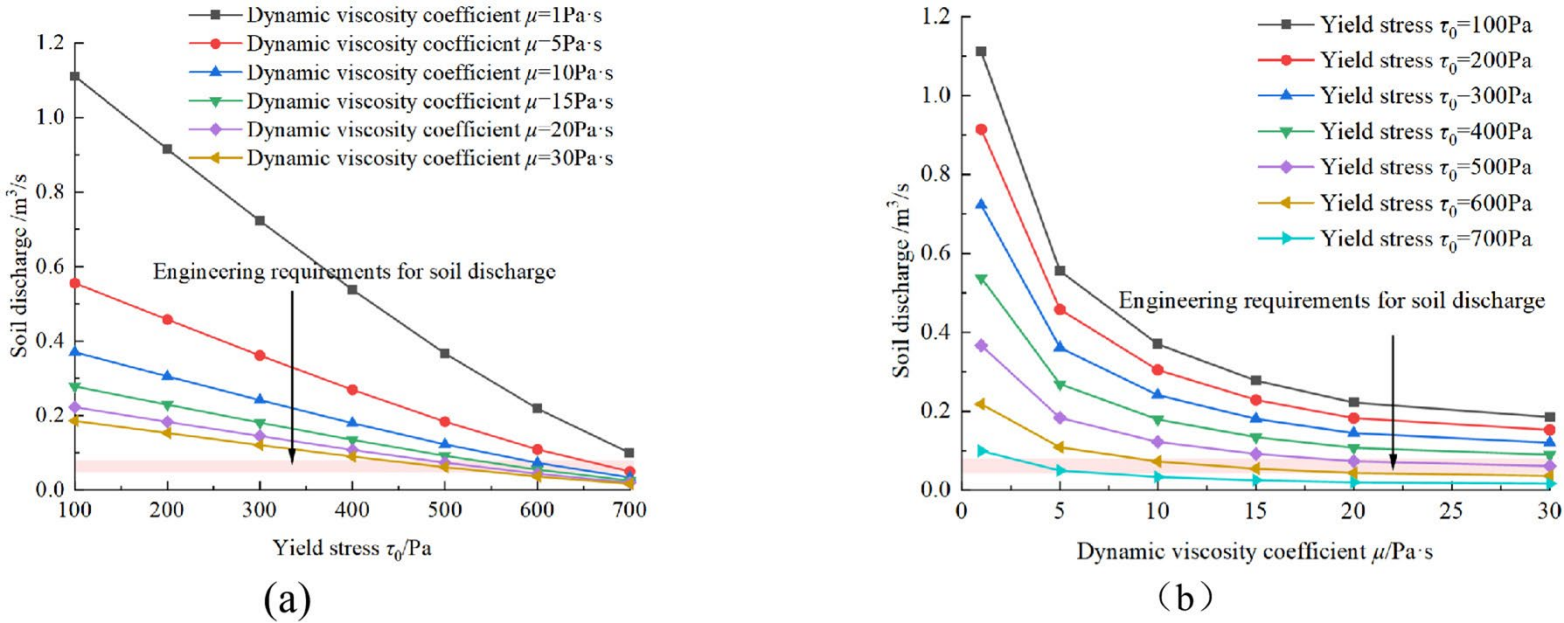
Influence of soil plastic flow on soil discharge under the 300 kPa pressure.

Figure 19 illustrates the influence of soil plastic flow (yield stress and dynamic viscosity coefficient) on the soil discharge. It can be seen that the soil discharge shows a linear decreasing trend with increasing yield stress, and a nonlinear decreasing trend with a gradient decline as the dynamic viscosity coefficient rises. As the yield stress of the soil increases, the pressure driving the flow of the soil needs to overcome greater frictional resistance, at the same time, the radius of the flow core grows larger, which causes the flow velocity of the soil to decrease. When the dynamic viscosity coefficient of soil increases, it means that under the same shear rate, the internal friction force given by the surrounding soil increases, and the flow velocity decreases accordingly. Therefore, it can be generally considered that the larger the rheological parameters of the soil the more difficult to discharge the soil.

**Fig. 20:**
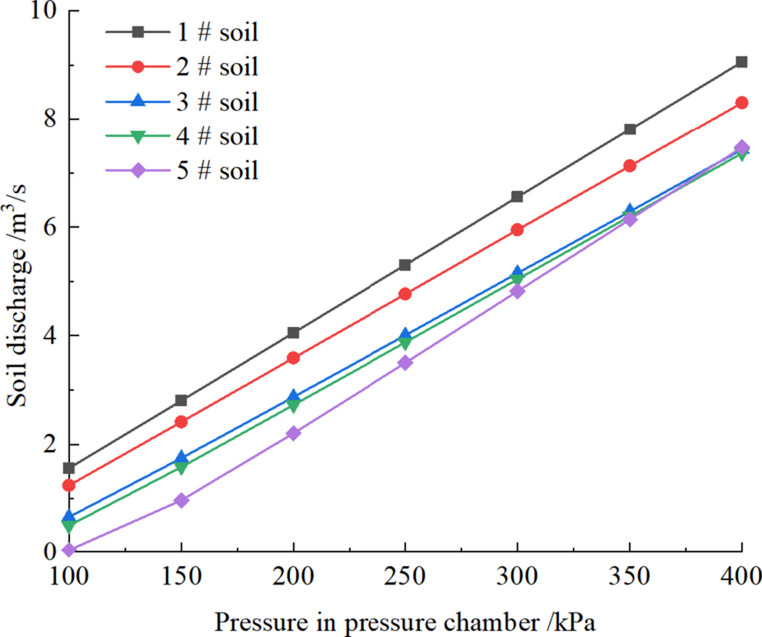
Influence of pressure on soil discharge.

illustrates the influence of pressure in pressure chamber on the soil discharge. It can be seen that the soil discharge linearly increases with the pressure. As shown in Figs. 19 and 21, soil discharge is fast and cannot be controlled solely by the screw conveyor when the yield stress and dynamic viscosity coefficient of the conditioned soil are low. Conversely, when the yield stress and dynamic viscosity coefficient of the conditioned soil are excessively high (i.e., the yield stress 700 Pa and the dynamic viscosity coefficient of 30 Pa·s), the soil discharge is 0.0165 m^3^/s, which is far less than the required soil discharge in engineering practice. This causes difficulties in soil discharge, and a large amount of soil is retained in the pressure chamber, which leads to adverse construction phenomena such as mud cakes. Therefore, to achieve a balance between excavation and discharge, the conditioned soil in the pressure chamber must possess optimal plastic flow for various tunnel burial depths. In other words,, as the burial depth of tunnel increases, the plastic flow of the conditioned soil also needs to change accordingly by increasing yield stress or dynamic viscosity coefficient. To determine the optimal soil plastic flow, a three-dimensional relationship diagram between soil discharge and soil plastic flow is established, as shown in Fig. 21.

**Fig. 21:**
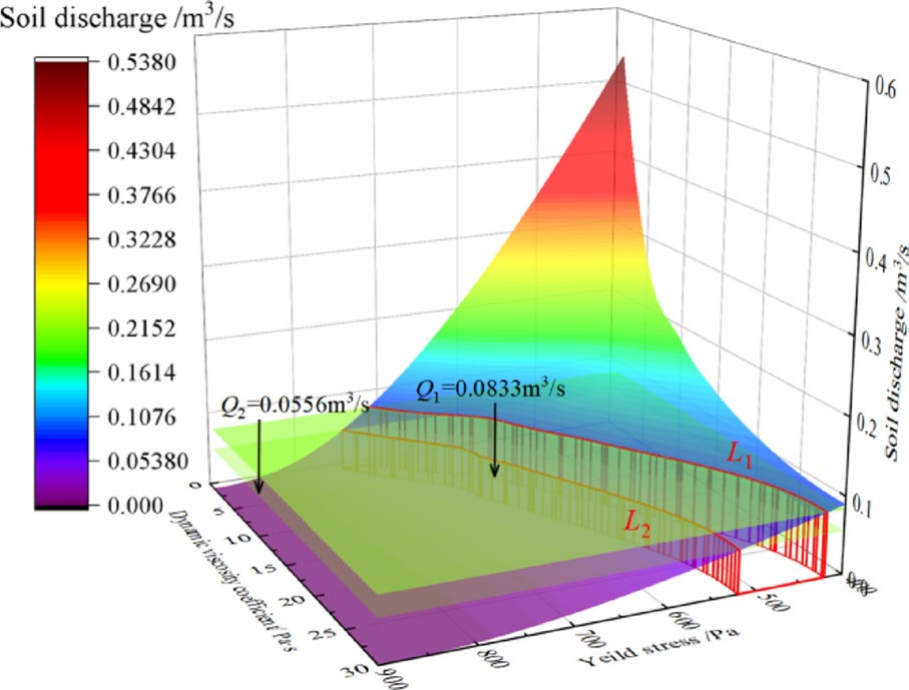
The influence of soil plastic flow on soil discharge.

**Fig. 22:**
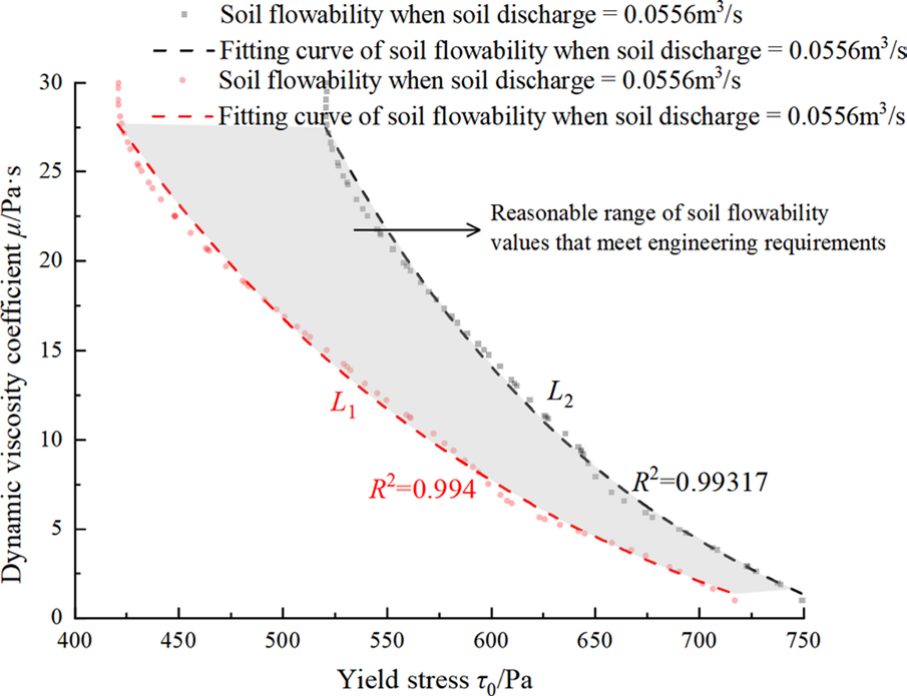
Reasonable plastic flow of conditioned soil.

From Fig. 21, it can be seen that the influence of yield stress and dynamic viscosity coefficient on soil discharge is basically consistent with Fig. 19. In order to determine the ideal plastic flow of the conditioned soil, *Q*_1_ = 0.0833m^3^/s and *Q*_2_ = 0.0556m^3^/s are respectively taken into Fig. 21, which intersects with the three-dimensional surface at *L*_1_ and *L*_2_. Therefore, these two intersecting lines are the critical values for the required soil discharge in the engineering practice. Then *L*_1_ and *L*_2_ are projected onto the *xy* - plane to obtain the Fig. 22, and the projection curves of *L*_1_ and *L*_2_ are fitted on the *xy* plane as follows:

21 $$\eqalign{ & {L_1}{\mu _1} = {\rm{ - 7}}{\rm{.403}} + {\rm{32}}{\rm{.923}}{{\rm{e}}^{{{{\rm{ - }}{\tau _0}{\rm{ - 434}}{\rm{.406}}} \over {213.11}}}} \cr & {L_2}{\mu _2} = {\rm{ - 7}}{\rm{.078}} + {\rm{32}}{\rm{.613}}{{\rm{e}}^{{{{\rm{ - }}{\tau _0}{\rm{ - 529}}{\rm{.8}}} \over {162.273}}}} \cr}$$

22 $$\mu_{1}\leq\mu_{2}\leq\mu_{3}$$

## Inverse analysis of soil plastic flow

### Inversion results

Based on the above analysis, it is evident that the soil discharge rate is related to the plastic flow of the soil, which is governed by the rheological parameters. However, the slump is only related to the yield stress. To determine a reasonable slump of conditioned soil, specific engineering cases need to be analyzed. Therefore, monitoring data from the shield tunneling construction between Hengli Station and Panyu Square Station on Guangzhou Line 18 are used as a reference. The objective function is constructed using the least squares method and satisfies the constraint condition in Eq. (22). Consequently, a constrained nonlinear least squares objective function was formulated as follows:

23 $$\begin{gathered} F(\mu , {\tau _0})=\frac{1}{2}\sum\limits_{{{\mathrm{i}}=1}}^{{\mathrm{m}}} {{{\mathrm{f}}_{\mathrm{i}}}( \mu , {\tau _0}{) ^2}} \hfill \\ {{\mathrm{f}}_{\mathrm{i}}}(\mu , {\tau _0})={Q_{\mathrm{i}}}{\mathrm{-}}{{\overset{\lower0.5em\hbox{$\smash{\scriptscriptstyle\frown}$}}{Q} }_{\mathrm{i}}},{\mathrm{i}}=0,1\, 2 \ldots \ldots m \hfill \\ \end{gathered}$$

Where $F(\mu , {\tau _0})$ is the objective function, $f_{i}(\mu , {\tau _0})$ is the loss function, $\hat Qi$ is the monitoring data of soil discharge, *Q*_i_ is the soil discharge calculated based on Eq. (19), and *m* is the number of monitoring data. In order to achieve the minimum value of the objective function $F(\mu , {\tau _0})$, it is required that the partial derivatives of $F(\mu , {\tau _0})$  for *u* and ${\tau _0}$  are all 0. For the convenience of expression, the following will refer to ${\tau _0}$ , *u* using *x*_1_ and *x*_2_:

24 $$\frac{{\partial F}}{{\partial {{\mathrm{x}}_{\mathrm{j}}}}}=\sum\limits_{{{\mathrm{i}}=1}}^{{\mathrm{m}}} {{{\mathrm{f}}_{\mathrm{i}}}} \frac{{\partial {{\mathrm{f}}_{\mathrm{i}}}}}{{\partial {{\mathrm{x}}_{\mathrm{j}}}}}=0( {\mathrm{j}}=1,2)$$

In the above nonlinear system, $\frac{{\partial F}}{{\partial {{\mathrm{x}}_{\mathrm{j}}}}}$ is a nonlinear function of variables and parameters, and the final solution cannot be obtained solely based on Eqs. (22) and (23). Therefore, it is necessary to give an initial value, and to approach the final value through iterative methods.

25 $${{\mathrm{x}}_{\mathrm{j}}} \approx {\mathrm{x}}_{{\mathrm{j}}}^{{{\mathrm{k}}+{\mathrm{1}}}}={\mathrm{x}}_{{\mathrm{j}}}^{{\mathrm{k}}}+\Delta {{\mathrm{x}}_{\mathrm{j}}}, {\mathrm{j}}=1, 2$$

Where *x*_*j*_ is the final value, *k* is the number of iterations, △*x*_*j*_ is the iteration step size, *f*_i_(*x*_*j*_^*k*+1^) was performed first-order Taylor expansion to improve computational efficiency:

26 $$\begin{gathered} {{\mathrm{f}}_{\mathrm{i}}}( {\mathrm{x}}_{{\mathrm{j}}}^{{{\mathrm{k}}+{\mathrm{1}}}}) \approx {{\mathrm{f}}_{\mathrm{i}}}( {\mathrm{x}}_{{\mathrm{j}}}^{{\mathrm{k}}}) +\frac{{\partial {{\mathrm{f}}_{\mathrm{i}}}}}{{\partial {{\mathrm{x}}_{\mathrm{j}}}}}({\mathrm{x}}_{{\mathrm{j}}}^{{{\mathrm{k}}+{\mathrm{1}}}}{\mathrm{-x}}_{{\mathrm{j}}}^{{\mathrm{k}}}) \hfill \\ ={{\mathrm{f}}_{\mathrm{i}}}( {\mathrm{x}}_{{\mathrm{j}}}^{{\mathrm{k}}}) +\frac{{\partial {{\mathrm{f}}_{\mathrm{i}}}}}{{\partial {{\mathrm{x}}_{\mathrm{j}}}}}\Delta {{\mathrm{x}}_{\mathrm{j}}} \Rightarrow \Delta {{\mathrm{x}}_{\mathrm{j}}}=\frac{{\Delta {{\mathrm{f}}_{\mathrm{i}}}}}{{\mathrm{J}}} \hfill \\ \end{gathered}$$

Where *J* is the Jacobian matrix, which is the partial derivative of the loss function over *x*_*j*_. So the partial derivative of the loss function for *u*, *τ*_0_ are as follows:

27 $${J_1}=\frac{{\partial {{\mathrm{f}}_{\mathrm{i}}}}}{{\partial {{\mathrm{x}}_1}}}=\frac{\pi }{{3\mu }}({\mathrm{r}}_{{\mathrm{e}}}^{{\mathrm{3}}} - {\mathrm{r}}_{{\mathrm{0}}}^{{\mathrm{3}}}) \:\:\:\:\:\: {J_2}=\frac{{\partial {{\mathrm{f}}_{\mathrm{i}}}}}{{\partial {{\mathrm{x}}_2}}}={\mathrm{-}}\frac{{\pi {\mathrm{r}}_{0}^{4}}}{{8{\mu ^2}}}\frac{{{\mathrm{dp}}}}{{{\mathrm{dx}}}}(1{\mathrm{-}}\frac{{\mathrm{4}}}{{\mathrm{3}}}\frac{{{{\mathrm{r}}_{\mathrm{e}}}}}{{{r_0}}}+\frac{1}{3}\frac{{r_{e}^{4}}}{{r_{0}^{4}}})$$

**Fig. 23:**
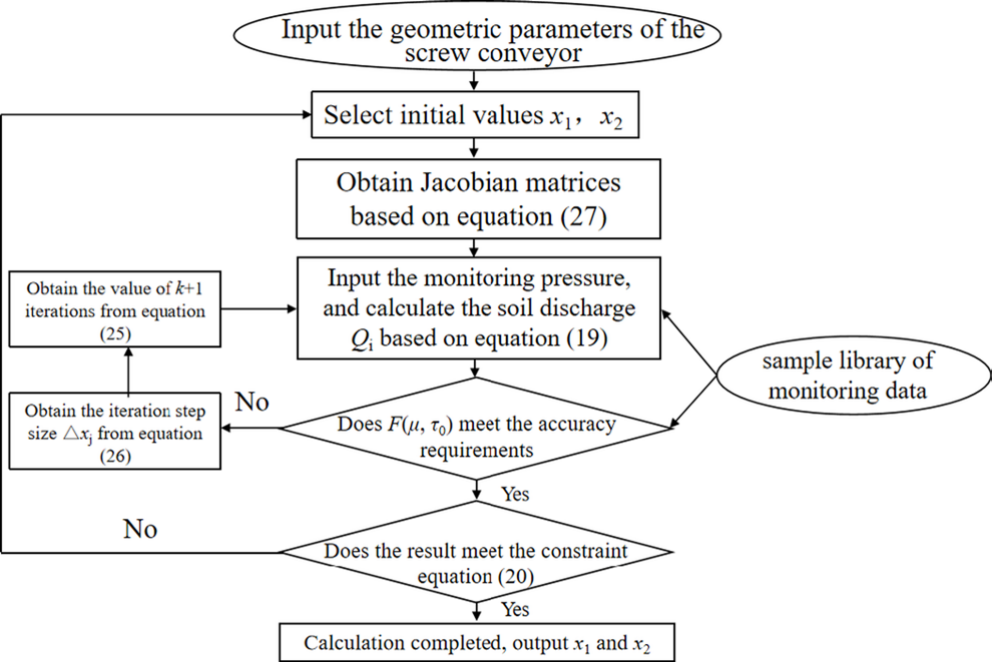
Inversion flowchart of soil plastic flow.

This paper established an inversion program based on Python, and the flowchart is illustrated in Fig. 23. The sample library in Fig. 23 is selected from the monitoring data on the right line of the shield tunnel from Hengli Station to Panyu Square Station. To ensure the accuracy of the inversion and justify the reliability of the sample library, raw data were processed to extract the stable excavation periods. The data filtering criteria specifically excluded the following:

Non-excavation periods: Data recorded during ring assembly, cutterhead maintenance, and other downtime.

Transient phases: The first and last 60 s of each excavation push, where parameters such as pressure and torque fluctuate significantly during startup and shutdown.

Static states: Periods where the screw conveyor speed was zero, which do not represent a continuous discharge process.

By applying these criteria, the “abnormal values” (noise caused by non-advancing operations) were removed, and a refined sample library representing steady-state shield tunneling was obtained, as shown in Table 3.

**Table 3:** .Sample library

Index of segment	Pressure in pressure chamber / kPa	Excavation time / min	Soil discharge / m^3^	Soil discharge per unit time /m^3^ / s	Burial depth of tunnel vault / m	Slump of soil/mm
1200	312	26	100	0.0641	31.1	80
1201	312	25	102	0.0680	31.1	82
1202	311	22	90	0.0682	31.2	81
1203	311	25	104.	0.0693	31.2	81
1204	312	30	99	0.0550	31.2	80
······						
1459	320	23	101	0.0733	31.6	79
1460	317	24	100	0.0695	31.6	85
······						
1686	315	26	98	0.0628	31.2	82
1687	310	25	100	0.0667	31.2	83
1688	310	24	102	0.0708	31.2	82

**Fig. 24:**
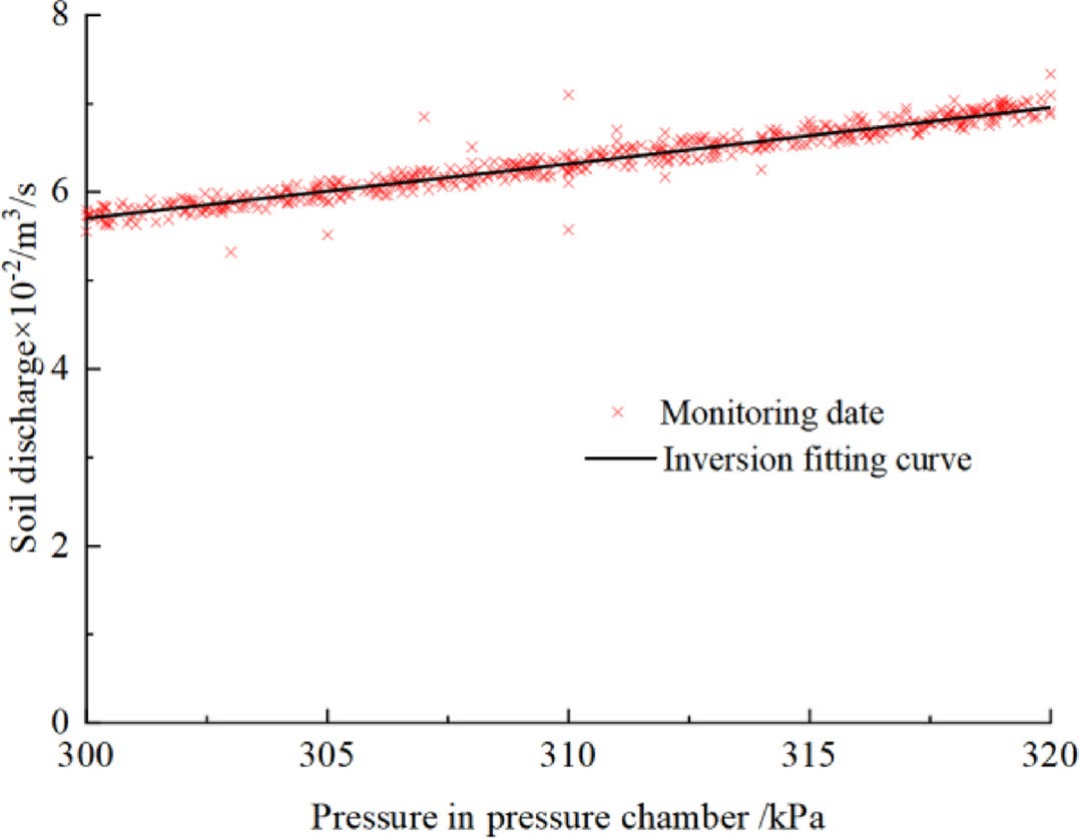
Inversion results of soil plastic flow.

Based on the inversion results shown in Fig. 24, the rheological parameters of the conditioned soil for this project are obtained: the yield stress is 526.5 Pa and the dynamic viscosity coefficient is 28.6 Pa·s. Substituting these parameters into Eq. (6) to (8), the corresponding slump is calculated to be 94.2 mm. In the actual project, the slump of the conditioned soil in this section of shield tunneling construction is approximately 80–90 mm. Considering the rotational discharge effect of the screw conveyor, it can be generally concluded that the inferred soil plastic flow is consistent with the soil plastic flow used in the actual project. This indicates that the inversion analysis is basically reliable.

From the above analysis, it is evident that the slump of the conditioned soil is related to the burial depth of the tunnel. As the burial depth of the tunnel increases, the slump of the conditioned soil should decrease accordingly to ensure the stability of excavation face. Based on Fig. 24, the ideal plastic flow of the conditioned soil at different tunnel burial depths can be further determined, and recommendations for conditioned soil slump can be provided. Since the calculated model satisfies the excavation and discharge balance condition, the static earth pressure can be used as the support pressure, which generally shows a linear relationship with the burial depth. According to Table 3, when the burial depth at the tunnel vault is approximately 30 m, the monitored pressure in the pressure chamber is generally in the range of 300 to 320 kPa. Therefore, it is not difficult to deduce the pressure in the pressure chamber at other burial depths. According to the excavation and discharge balance conditions of the EPB shield tunneling, the shield machine must ensure that the discharge volume equals the excavation volume. Therefore, for shield machines of the same size, the discharge volume remains constant even if the tunnel burial depth changes. To further explore the reasonable plastic flow of the conditioned soil for the 8.8 m shield under different burial depth, the sample library in Table 3 can continue to be used.

**Fig. 25:**
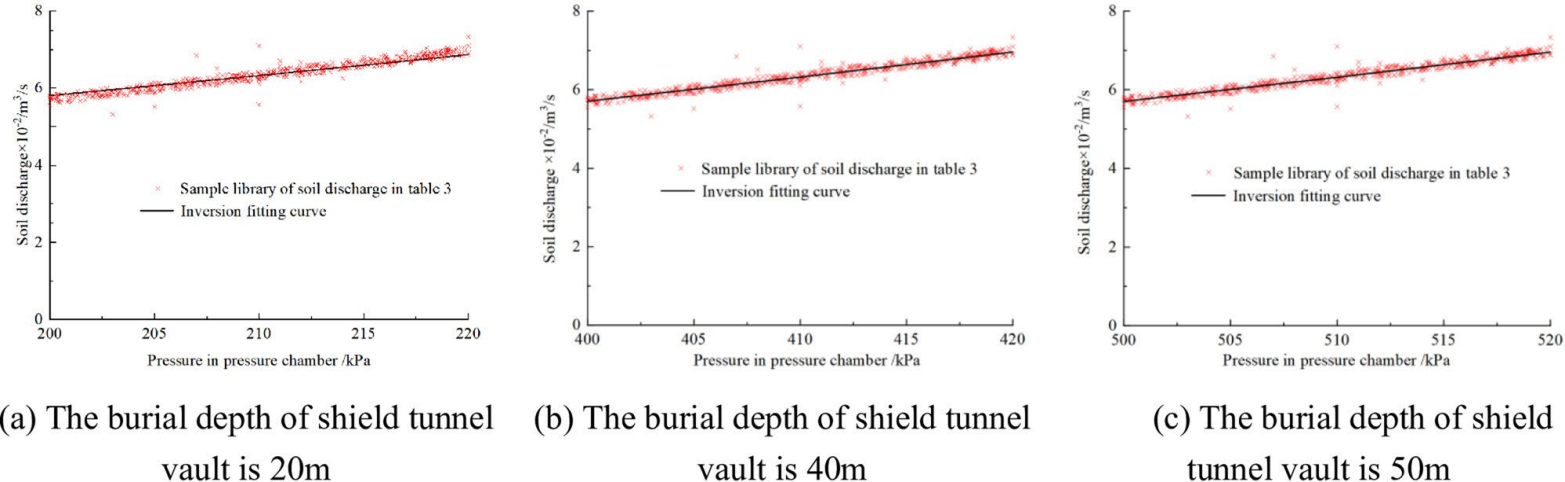
Inversion results of soil plastic flow under different tunnel burial depths. (**a**) The burial depth of shield tunnel vault is 20 m, (**b**) The burial depth of shield tunnel vault is 40 m, (**c**) The burial depth of shield tunnel vault is 50 m.

Figures 24 and 25 illustrate the reasonable plastic flow of the conditioned soil at different tunnel burial depths. When the burial depth of shield tunnel vault is 20 m, the yield stress of the conditioned soil should be 245.1 Pa, and the dynamic viscosity coefficient should be 33.4 Pa·s; When the burial depth of shield tunnel vault is 30 m, the yield stress of the conditioned soil should be 526.5 Pa, and the dynamic viscosity coefficient should be 28.6 Pa·s; When the burial depth of shield tunnel vault is 40 m, the yield stress of the conditioned soil should be 689.1 Pa, and the dynamic viscosity coefficient should be 37.3 Pa·s; When the burial depth of shield tunnel vault is 50 m, the yield stress of the soil should be 901.2 Pa, and the dynamic viscosity coefficient should be 37.8 Pa·s. Substituting the reasonable plastic flow of the conditioned soil obtained from the above inversion into Eq. (6) to (8), the suggested slump of the conditioned soil can be obtained: when the burial depth of shield tunnel vault is 20 m, the slump of the conditioned soil should be 177 mm; When the burial depth of shield tunnel vault is 30 m, the slump of the conditioned soil should be 94 mm; When the burial depth of shield tunnel vault is 40 m, the slump of the conditioned soil should be 60 mm; When the burial depth of shield tunnel vault is 50 m, the slump of the conditioned soil should be 28 mm.

### Engineering discussion

The inversion analysis and subsequent depth-dependent study provide critical insights into soil conditioning, yet the practical application requires a nuanced understanding of measurement discrepancies and functional priorities.

1) Analysis of Slump Discrepancies Although the inverted slump (94.2 mm) is consistent with the general trend of field records (80–90 mm), a discrepancy of approximately 5–15 mm exists. This is primarily attributed to sampling delay and foam degradation. Field slump tests are conducted at the discharge gate, several minutes after the soil leaves the pressurized chamber. During this interval, foam maturation and bubble collapse (governed by the half-life) lead to a reduction in fluidity. Additionally, while the model assumes an average chamber state, samples from the screw conveyor may be slightly denser due to mechanical disturbance. Thus, the theoretical value reflects the “in-situ” state within the chamber, while field measurements reflect the slightly “stiffer” state after discharge.

2) Priority Shift: Sealing vs. Flowability The study indicates that for deep tunnels (e.g., 50 m burial depth), the suggested slump drops to an extremely low value of approximately 28 mm. While such a stiff, clay-like consistency may increase cutterhead torque and mechanical wear, it serves a vital engineering purpose: the formation of an impermeable “earth plug”. In high-water-pressure environments, the conditioned soil must prioritize its water-sealing function and anti-permeability to resist the high hydrostatic pressure gradient within the screw conveyor.

3) Trade-offs in Deep Excavation In such scenarios, maintaining an ideal flowability becomes secondary to preventing controlled discharge or “spewing.” The use of PAM and fine particles as conditioners is essential here, as they provide the necessary cohesion to form a stable plug while maintaining enough lubrication to prevent mechanical jamming. Recognizing this requirement for a low-slump, high-sealing matrix provides a theoretical threshold for engineers to balance the trade-off between operational torque and anti-spewing safety in deep-buried EPB shield tunneling.

## Conclusion

In this paper, the conditioned soil is assumed to be an ideal Bingham fluid. Based on the rheological theory, the relationship between the rheological parameters of the conditioned soil and the slump is derived, and a mechanical model for the soil discharge is established. Finally, using the monitoring data from the shield tunneling construction between Hengli Station and Panyu Square Station on Guangzhou Line 18, the ideal rheological parameters and suggested slump of the conditioned soil are inverted under different tunnel burial depths. The following conclusions are drawn:

1. In the soil conditioning scheme that incorporates fine particles with PAM, the polymer chains of PAM establish a three-dimensional cross-linked network among the fine particles, thereby enhancing the stability of the conditioned soil. This network enables the conditioned soil to achieve favorable plastic flow at any slump, and its rheological properties essentially meet the rheological constitutive behavior of a Bingham fluid.
2. The soil discharge rate is controlled by the pressure in the pressure chamber, the yield stress, and the dynamic viscosity coefficient. As an increase in the pressure and yield stress, the soil discharge rate increase and decrease linearly, respectively. Moreover, the soil discharge rate decreases nonlinearly as an increase in the dynamic viscosity coefficient increases.
3. Based on the requirements of the soil discharge in the section between Hengli Station and Panyu Square Station on Guangzhou Line 18, the ideal range of soil plastic flow that meets the balance of soil excavation and discharge in the pressure chamber are obtained. By using the monitoring data to perform inverse analysis, the optimal slump of conditioned soil under different tunnel burial depths were determined. When the burial depths of tunnel are 20, 30 and 40 m, the suggested slumps of conditioned are 177, 94 and 60 mm, respectively.
